# Introducing the 2024 THT Shark Tank Edition of *JACC: Basic to Translational Science*

**DOI:** 10.1016/j.jacbts.2024.03.002

**Published:** 2024-04-22

**Authors:** Douglas L. Mann

**Figure:**
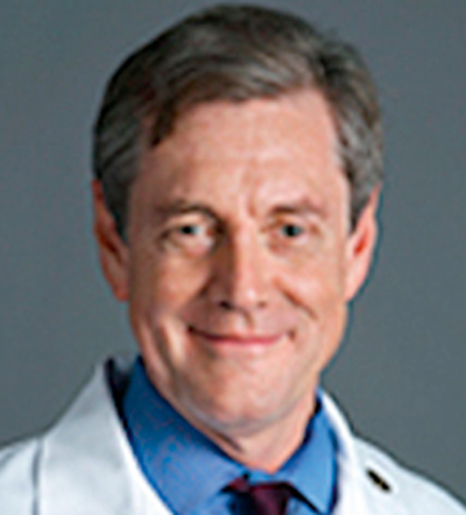

The global socioeconomic burden imposed by heart failure (HF) continues to rise unabated. Despite significant advancements in medical and device therapies, the symptoms of HF generally worsen over time, and most patients ultimately will die secondary to the complications arising from HF. The Technologies and Heart Failure Therapeutics (THT) meeting was created to showcase innovation and celebrate those behind exciting new efforts to manage and treat HF patients (https://tht2024.crfconnect.com/). On behalf of the editors of *JACC: Basic to Translational Science*, I am delighted to introduce the 2024 Technology and Heart Failure Therapeutics (THT) Shark Tank Edition. Building on the success of the past 2 year's collaboration with the Cardiovascular Research Foundation, we are dedicating a significant portion of the April issue to highlight the innovative technologies showcased in the 2024 THT Shark Tank program held in Boston, Massachusetts, on March 5, 2024.

In this issue, we present research letters summarizing the presentations made by startup companies during the third annual THT Shark Tank competition. Each presentation is accompanied by a video link featuring the inventors and a discussion of the presentations by the sharks. The focus issue covers a range of technologies shown in the following text, including monitoring devices and devices to manage HF with reduced ejection fraction (HFrEF) and preserved ejection fraction (HFpEF).

• Transcatheter Pulmonary Artery Banding for HFrEF: Initial Results, Exercise Hemodynamics From the Ongoing First-In-Human Trial View the presentation
• Heart Failure Solutions: The PeriCut Device as a Treatment for HFpEF View the presentation
• Pulsatile Ventricular Assist Platform: A Novel Surgically Implanted Ventricular Assist Device View the presentation
• NIHA-HF: An Artificial Intelligence Solution for Heart Failure Diagnosis and Monitoring Based on Lead I Electrocardiogram View the presentation
• Pulsatile ECMO: The Future of Mechanical Circulatory Support for Severe Cardiogenic Shock View the presentation

I trust that our readers will share my perspective that we are on the cusp of introducing innovative new strategies that are poised to enhance the treatment outcomes for individuals afflicted with HF. I would be remiss if I failed to express my gratitude to Dr Dan Burkhoff alongside our editorial team for ensuring the timely publication of this special issue just a month following the THT conference. My thanks extend to Stephanie Gutch, Senior Director of TCTMD/Digital at the Cardiovascular Research Foundation, for her invaluable assistance in integrating video content from the THT conference onto the *JACC* website. My appreciation also goes out to the exceptional *JACC* team for making this publication a reality, including, but not limited to, Justine Turco (Divisional Vice President, Scientific Publications & Guidelines, American College of Cardiology), Monica Payne-Emerson (Executive Managing Editor for the *JACC* journals), with a special shoutout to Kim Trevey (Managing Editor) and Jennifer Rapp (Editorial Assistant), whose remarkable efficiency and commitment to publishing the Shark Tank issues are truly indispensable.

As always, I look forward to receiving your comments on this special edition of *JACC: Basic to Translational Science*, whether it be through social media using the hashtag #JACCBTS or via email at JACC@acc.org.

